# Robotic-assisted vs non-robotic traction techniques in endoscopic submucosal dissection for malignant gastrointestinal lesions

**DOI:** 10.3389/fonc.2022.1062357

**Published:** 2022-10-25

**Authors:** Zhao Meng, Zhanghua Huang, Bingli Deng, Liuming Ling, Yaowen Ning, Shoaib Mohammed Rafiq

**Affiliations:** ^1^ Department of Gastroenterology, The Second People’s Hospital of Qinzhou, Qinzhou, Guangxi, China; ^2^ Department of Emergency and Trauma Center, District Headquarter Teaching Hospital, Gujranwala, Pakistan

**Keywords:** endoscopic submucosal dissection, robotic assisted traction, non-robotic traction, endoscopic traction, gastrointestinal cancer

## Abstract

Endoscopic submucosal dissection is an effective approach with higher en bloc resection and complete resection rate for superficial gastrointestinal (GI) lesions. However, endoscopic submucosal dissection is technically challenging and associated with several adverse events, such as bleeding or perforations. The single channel flexible endoscope’s intrinsic limitations in preserving visualization of the submucosal dissection plane as compared to laparoscopic surgery are the most common cause of complications during the endoscopic submucosal dissection technique. As a result, traction techniques were created as the endoscope’s second helping hand in order to improve the effectiveness of the endoscopic submucosal dissection method. Trainees can master endoscopic submucosal dissection methods more quickly by using traction techniques. The anatomical location of the lesion plays a major role in determining which traction technique should be employed. An appealing way of traction is robot-assisted endoscopic submucosal dissection, and various types of endoscopic robots that allow bimanual operation are currently being developed. The advent of robot-assisted endoscopic technology ushers in a new era of endoscopic submucosal dissection, and with it come its own unique challenges that remain to be elucidated. Future research and development efforts are needed to focus on pathways and curriculums for trainees to master the currently available traction techniques and provide avenues for the development of newer traction modalities. In this article, we discuss evolution, characteristics, technological improvements and clinical comparisons of both robotic and non-robotic endoscopic traction techniques used in endoscopic submucosal dissection.

## Introduction

Endoscopic submucosal dissection (ESD) has become the widely accepted and minimally invasive therapy of choice for superficial gastrointestinal (GI) tumors (1–3). ESD is an efficient therapeutic endoscopic procedure with a high en bloc resection rate and lower local recurrence rate compared to endoscopic mucosal resection (4, 5). However, ESD is technically challenging because it is associated with prolonged procedure time and several adverse events. The most common complications of ESD are perforation and bleeding (6, 7). In addition, it also requires stricter access conditions and longer learning curve than endoscopic mucosal resection which limits the worldwide popularity, especially in the United States and Western Countries (8, 9).

The major cause of complications in ESD procedure is that the visibility of the dissection plane is not secured because of the mucosal flap (10). Appropriate tissue tension and clear visibility of the tissue to be dissected using traction are essential for effective and safe ESD procedure. Although, the use of a transparent cap and reduplicative submucosal injection before the next resection is beneficial to obtain clear visibility of the submucosal layer. However, the supporting capacity of cap is limited and repeated injections prolong the procedure time. Another simple method is to change the patient’s position during the procedure for adequate tissue tension, but when the lesion is in the upper GI tract, the optimal position is limited. Although expert and experienced endoscopists can perform the endoscopic resection using superior dexterity gained from rich experience, unskilled endoscopists commonly find it technically demanding and difficult to complete the resection without good visibility of the dissection plane. The lack of a controllable second hand in ESD is a major difference compared with laparoscopic surgery which has three ports to assist the procedure.

Inspired by the surgical pull and push techniques, traction technology arisen and was applied as a “second-hand” for endoscope (11). With the assistant of some accessories, it can not only provide a clear view of submucosal tissue and vessel for operation but also gets adequate tissue tension, which facilitates ESD to be more effective and safer. In recent years, traction techniques have developed rapidly, including clip-with-snare traction, clip-with-line traction, magnetic anchor traction, percutaneous traction, external forceps traction, and internal-traction method (12–15). Although, these techniques are effective for simple counter traction, but they are still restricted in terms of being able to regulate the direction of the traction, change the submucosal layer’s tension, and re-grasping of the tissue. Some of these techniques are also invasive and only effective in certain regions of the GI tract. Endoscopic use of flexible robotic arms may aid the operator in getting over the ESD procedure’s technical challenges (16). Robot-assisted ESD is a desirable traction technique, and several kinds of endoscopic robots that permit bimanual operation are currently being developed. A new era of endoscopic surgery with robot assistance has begun. The goal of this study is to compare robotic and non-robotic endoscopic traction techniques in endoscopic submucosal dissection, as well as to review their development and characteristics.

## Non-robotic endoscopic traction

### Double-scope technique

In the double-scope traction technique, a second small-caliber endoscope is inserted along with the main endoscope after circumferential resection. By passing a share, net, or forceps through the second endoscope’s channel, and deploying the instrument of interest on the desired edge of tissue undergoing resection, traction can be applied (17). The primary endoscope is responsible for ESD. The double-scope method is not feasible for deep intubation of the GI tract, and hence is generally used for colorectal or gastric ESD (18). Additional limitations of the double-scope method include friction from the two endoscopes, requirement of two endoscopists, and difficulty in resection of large lesions due to insufficient space for maneuvering and operability.

### Double-channel scope traction (the R-Scope)

The double-channel endoscope (also known as the R-scope; Olympus) has two movable instrument channels: one moves grasping forceps vertically for lesion counter traction; the other swings a cutting knife horizontally for dissection (19, 20). The R-scope is heavier and more difficult to operate than a single-channel endoscope. Maneuvering two tools simultaneously through the double-channel-scope is technically challenging and time-consuming. The learning curve for efficient use and troubleshooting common problems that one may face while using the R-scope also needs to be elucidated.

## Robot-assisted traction techniques

### EndoSamurai

The EndoSamurai (Olympus Medical Systems Corp, Tokyo, Japan) consists of an endoscopic shaft and two independent arms with built-in working channels for interchanging surgical tools (21, 22). These independent arms are parallel to the endoscope shaft and can be opened after introduction of the scope to the site of interest. A third working channel is available within the endoscopic shaft. However, this working channel allows relatively less triangulation compared to the two independent arms, which translates into suboptimal tissue counter traction. The EndoSamurai system requires an overtube for the insertion of the scope, and two endoscopists are needed for operation, (one at the scope and one controlling the command console for the two working channel arms. The EndoSamurai system allows five degrees of freedom. The complexity of controlling a multi-channel therapeutic endoscope needs further study, as does elucidation of a learning curve. Additionally, retraction of larger organs can be difficult which limits the potential for resection with clear margins. Furthermore, no human studies exist to date and the feasibility of this system *in vivo* remains to be seen.

### ANUBIScope

The ANUBIS project was a result of collaboration between Storz and Institut de Recherche contre les Cancers de l’Appareil Digestif (IRCAD). The ANUBIScope consists of a four-way articulating flexible endoscopic shaft with two “wings” which are closed during introduction of the scope, acting as a tulip shaped blunt trocar to prevent luminal injury (23, 24). These “wings” house two 4.2 mm working channels, whereas a third 3.4 mm working channel is housed in the endoscope shaft. Similar to the EndoSamuai system, tissue retraction in a retroflexed position has been reported to be difficult with the ANUBIScope and remains an area of further experimentation. Additionally, the ANUBIScope requires cooperation and synchronized workflow between two physicians. The STRAS/ANUBIScope system is a robotic version of the ANUBIScope which has a tele-operated interface that obviates the need for a second physician and has been successfully used to perform ESD in porcine models with a favorable safety profile. Nonetheless, further study is needed before adoption of this system in human subjects.

### Master and slave trans-endoluminal robot

The MASTER system consists of a master controller, a telesurgical workstation to independently control endoscopically deployed surgical tools for ESD, and a custom-designed therapeutic endoscope with two working channels. It should be performed by two operators, the endoscopist maneuvering the endoscope and the surgeon controlling the master robotic controller. Gastric ESD assisted by MASTER has been reported in several studies (25, 26). The MASTER system has been tested for EFTR as well as NOTES hepatic wedge resection with favorable preliminary results in terms of procedure time, maneuverability, degrees of freedom, ease of use and the cognitive load while performing procedures. More recently, a randomized, controlled, ex vivo study comparing conventional ESD to robot-assisted ESD (RESD) showed that RESD resulted in a higher en bloc resection rate with a shorter procedure time and a lower perforation rate. Robotic traction techniques, however, are currently in their infancy, and further experimental studies are needed to explore the safety and efficacy profile of these modalities.

## Conclusion and future perspective

Endoscopic submucosal dissection is a landmark technique in the development of endoscopic therapy and has been rapidly promoted. Although endoscopic submucosal dissection is an effective therapy for superficial lesions in the GI tract, it is challenging and requires a high degree of skill levels, which might hinder its execution and raise the possibility of complications (27, 28). The current ESD procedure carries the high risk of complications such as bleeding and perforation resulting from blind dissection. This is because a standard endoscope has inherent limitations in maintaining visualization of the submucosal dissection plane due to its one-handed operation capacity. A long period of training is required for endoscopists because they must undergo extensive training before they can perform ESD in a secure and expert manner. Variety of non-robotic traction techniques have been emerged to overcome these limitations with the goal of achieving proper counter traction. These traction techniques can achieve good, clear visualization of the submucosal layer using only a few, easy-to-configure components. However, they are constrained by the fact that the direction of traction cannot be changed, and the modification process is difficult because re-grasping is not allowed (29–31). Moreover, the tension of traction can reduce over time in some of these techniques. Robot-assisted traction in endoscopic submucosal dissection is another attempt to mitigate this difficulty.

Various endoscopic robot systems with twin arms that can be operated bimanually are currently available in the market. Robotic systems have shown success in therapeutic endoscopic operations, but the development of specially tailored endoscopes and tools had delayed their widespread clinical use. According to several investigations, it is theoretically possible to do endoscopic submucosal dissection using a simple robot that enables the endoscopist to dynamically apply counter traction. In comparison to conventional non-robotic procedures, robotic-assisted traction could significantly shorten the duration of the surgery and obtain a greater rate of direct vision dissection. Robotic-assisted traction is simple to implement into clinical practice and decreases the learning curve of endoscopic submucosal dissection for beginners. Additionally, it enables the endoscopist to carry out standard endoscopic submucosal dissection. Consequently, further research into the robotic-assisted traction strategy is necessary.

## Authors contributions

Study concept and design: ZM, ZH. Manuscript writing: ZM, ZH, SR. Collection, analysis and interpretation of data: BD, LL, YN. Critical revision of manuscript: ZM, SR. All authors contributed to the article and approved the submitted version.

## Conflict of interest

The authors declare that the research was conducted in the absence of any commercial or financial relationships that could be construed as a potential conflict of interest.

## Publisher’s note

All claims expressed in this article are solely those of the authors and do not necessarily represent those of their affiliated organizations, or those of the publisher, the editors and the reviewers. Any product that may be evaluated in this article, or claim that may be made by its manufacturer, is not guaranteed or endorsed by the publisher.
